# Efficacy and Safety of Cell-Assisted Acellular Adipose Matrix Transfer for Volume Retention and Regeneration Compared to Hyaluronic Acid Filler Injection

**DOI:** 10.1007/s00266-024-04408-0

**Published:** 2024-10-01

**Authors:** Xian Jin, Hyokyung Yoo, Vinh Vuong The Tran, Chenggang Yi, Ki Yong Hong, Hak Chang

**Affiliations:** 1 Present Address: Department of Plastic Surgery, The Second Affiliated Hospital of Zhejiang University School of Medicine, Hangzhou, China; 2 Department of Plastic and Reconstructive Surgery, Seoul National University Hospital, Seoul National University College of Medicine, 101 Daehak-ro, Jongno-gu, Seoul, 03080 Republic of Korea; 3 Department of Plastic and Reconstructive Surgery, Seoul National University College of Medicine, Seoul, Republic of Korea

**Keywords:** Acellular adipose matrix, Adipose-derived stem cells, Soft tissue augmentation, Volume retention, Scaffold

## Abstract

**Background:**

Cell-assisted acellular adipose matrix (AAM) transfer is a novel technique for soft tissue volume restoration, where AAM acts as a scaffold for tissue proliferation and promotes host cell migration, vascularization, and adipogenesis. This study aimed to evaluate the efficacy and safety of in vivo cell-assisted AAM transfer compared to hyaluronic acid (HA) filler injection.

**Methods:**

Human adipose tissue was used to manufacture AAM, and murine adipose-derived stem cells (ASCs) were prepared. Nude mice were divided into four groups: AAM transfer (AT), ASC-assisted AAM transfer (CAT), HA filler injection (HI), and ASC-assisted HA filler injection (CHI). Eight weeks post-transfer, in vivo graft volume/weight, histology, and gene expression were analyzed to assess efficacy and safety.

**Results:**

The AAM retained its three-dimensional scaffold structure without cellular components. AT/CAT showed lower volume retention than HA/CHA; however, CAT maintained a similar volume to HA. Histologically, adipogenesis and collagen formation were increased in AT/CAT compared to HA/CHA, with CAT showing the highest levels. CAT also demonstrated superior angiogenesis, adipogenesis, and gene expression (Vegf and Pparg), along with lower Il-6 expression, higher Il-10 expression, and reduced capsule formation, indicating better biocompatibility.

**Conclusions:**

Cell-assisted AAM transfer is a promising technique for volume retention and tissue regeneration, offering a safe and effective alternative to HA filler injections.

**Level of Evidence III:**

This journal requires that authors assign a level of evidence to each article. For a full description of these Evidence-Based Medicine ratings, please refer to the Table of Contents or the online Instructions to Authors www.springer.com/00266.

**Supplementary Information:**

The online version contains supplementary material available at 10.1007/s00266-024-04408-0.

## Introduction

Hyaluronic acid (HA) filler injection and autologous fat grafting are the most frequently used methods for reconstructing small soft tissue defects. Although filler injection is a convenient tool to restore volume for various reconstructive and cosmetic purposes, it does not replicate all characteristics of living tissues and can induce foreign body-related complications, infection, necrosis, or granuloma formation. Autologous fat grafting provides biological soft-tissue replacement and lowers the risk of foreign body-related complications. However, unpredictable rates of fat resorption, necrosis, calcification, oil cyst formation, limited donor availability, and comorbidities related to donor sites persist as limitations [1–3].

Recently, acellular adipose matrix (AAM), a decellularized form of bioscaffold obtained from discarded fat tissue, has attracted considerable attention because of its wide range of sources and good volume repair capacity [4]. Large amounts of fat tissue from various surgical procedures can be recycled for regenerative medicine. AAM is advantageous regarding better biocompatibility and lower cost than HA. It is less invasive, and has a higher predictability of graft survival than autologous fat grafting [3]. AAM is composed of a structural network of proteins and proteoglycans with viscoelastic properties similar to those of lipoaspirates, suggesting it as an ideal candidate for soft tissue augmentation [5]. Moreover, AAM not only serves as a scaffold for tissue regeneration but also plays a profound role in cellular processes, including cell migration, proliferation, differentiation, and apoptosis [6]. The concept of combining stem cells with AAM is called cell-assisted AAM, in which the supplemented stem cells are known to promote vascularization and tissue regeneration [7].

However, biomaterial derived from native adipose tissue is currently not approved by the Food and Drug Administration due to the lack of clinical evidence of its efficacy and safety [8]. Most of the relevant studies are still in preclinical stages. Although acellular dermal matrices with similar concepts are widely used in various surgical fields and have a huge global market, the use of AAM is very limited. This study aimed to verify the efficacy and safety of the in vivo transfer of cell-assisted AAM and its potential as an off-the-shelf commercial alternative to HA fillers.

## Methods

### Study Design

A modified protocol for AAM manufacturing, encompassing both physical and chemical interventions, was established. Subsequently, in vitro cytotoxicity tests were conducted to evaluate biocompatibility. In animal studies, HA filler and AAM groups with or without adipose-derived stem cell (ASC) supplementation were compared. The rates of in vivo volume retention, vascularization, adipogenesis, inflammation, and capsule formation were evaluated by histology, immunohistochemistry (IHC), and gene expression analyses. The safety of AAM has recently been demonstrated in clinical settings involving human participants. However, both preclinical and clinical investigations have not successfully achieved substantial adipose tissue formation within AAM *in vivo* [9, 10]. Notably, adipogenesis has primarily been identified in the peripheral regions of AAM transfer, which may not fully meet the clinical standards for soft tissue reconstruction. To address this limitation, we incorporated an ASCs-enriched experimental group aimed at improving the efficiency of *de novo* adipogenesis within AAM, thereby enhancing its potential as a biomaterial [11]. The study utilized human adipose tissue to create AAM and murine adipose-derived stem cells (ASCs). Twenty-four nude mice were randomized into four groups: AAM transfer, ASC-assisted AAM transfer, HA filler injection, and ASC-assisted HA filler injection. Each group contained of six mice, based on power calculations ensuring 80% statistical power. Rigorous randomization and group allocation concealment minimized bias, while statistical tests confirmed group comparability. Blinded assessment methods were employed during data collection and analysis to further enhance the integrity of the findings.

### AAM Manufacture and Evaluation

#### Preparation of AAM

Human adipose tissue was obtained from a female donor who had undergone breast reconstruction using a free transverse rectus abdominis myocutaneous flap at our institute in 2022. Subcutaneous fat (100 mL) was obtained from the remaining portion of the harvested flap after inset. This study was approved by the Institutional Review Board (No. 2209-106-1359) and performed in accordance with the recommendations of the Declaration of Helsinki for biomedical research involving human subjects. Written informed consent was obtained from the study participant.

**Fig. 1:**
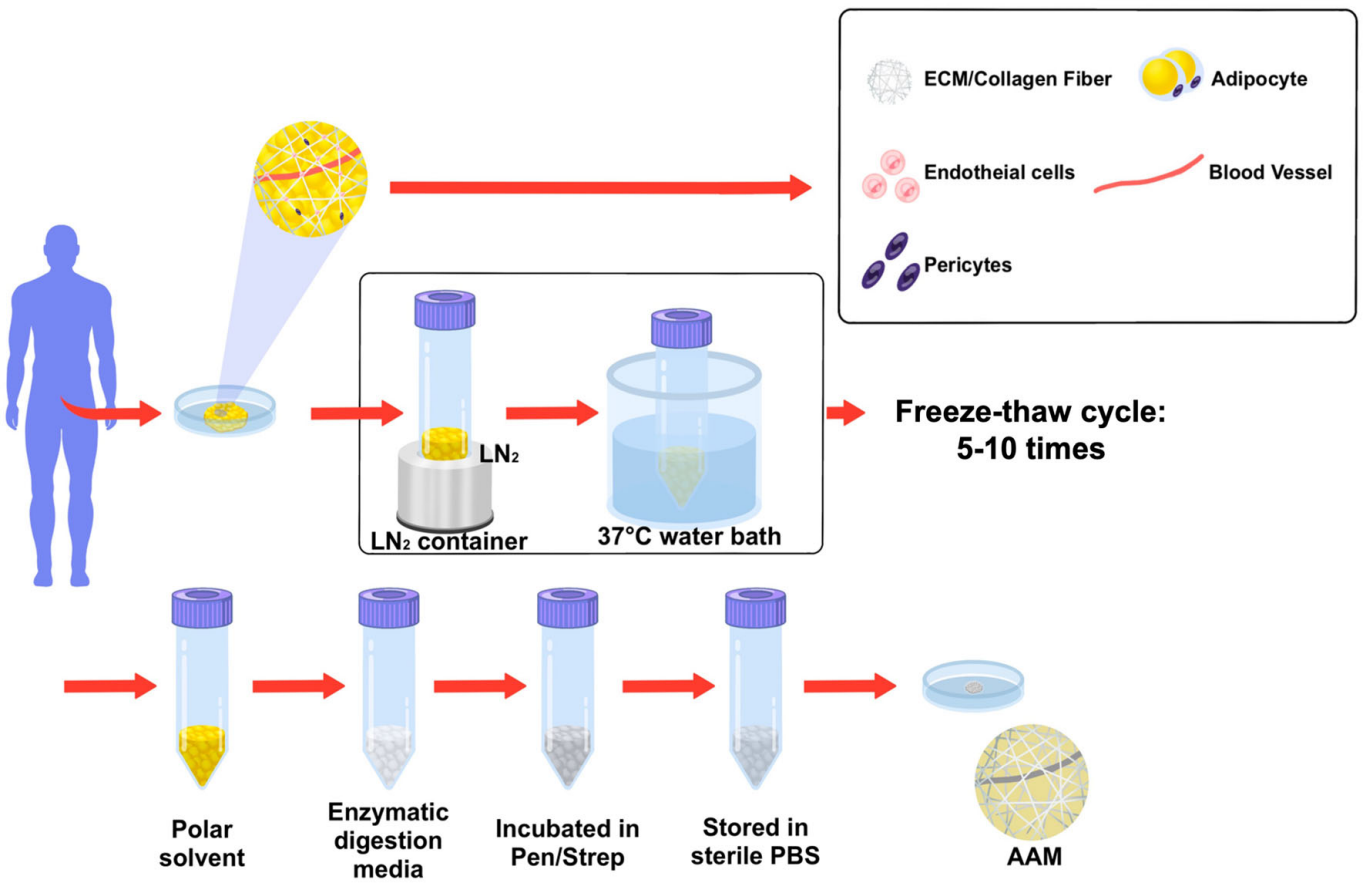
Schematic representation of the manufacture of AAM. AAM was manufactured from human adipose tissue through mechanical, chemical, and enzymatic processes. The freeze-thaw cycle was repeated up to 10 times to minimize the following chemical treatments. *AAM* acellular adipose matrix, *ECM* extracellular matrix, *LN2*: liquid nitrogen, *PBS* phosphate-buffered saline, *Pen* penicillin, *Strp* streptomycin

A modified version of a previously published AAM manufacturing protocol [12, 13] was used (Fig. 1). The adipose tissues were submersed in liquid nitrogen for 10 min and immediately placed in a 37 °C water bath for 15 min. This freeze-thaw cycle was repeated up to ten times to maximize the mechanical destruction of tissues and to minimize the following chemical procedures to improve biocompatibility. The tissues were centrifuged at 1,500 rpm for 10 min and washed with phosphate-buffered saline (PBS), and the upper fatty liquid portion was selectively removed. The remaining tissues were subjected to polar solvent extraction using 99.9% isopropanol solution for 8 h to eliminate the lipid content. Then, the processed tissues were rinsed in PBS again and incubated in enzymatic digestion medium, composed of 0.05% trypsin, 0.05% EDTA, 10 ng/mL DNAse I (Merck KGaA, Darmstadt, Germany), and 10 ng/mL RNAse (Merck KGaA), for 2 h with slow rotation in a 37 °C incubator to completely remove the cellular components. Following PBS washing, the tissues were incubated in 1% penicillin and streptomycin (both Merck KGaA) for 12 h at 4 °C and stored in PBS at 4 °C.

#### Confirmation of the Manufactured AAM

The samples of fresh human adipose tissue and manufactured AAM were fixed in 2.5% glutaraldehyde for 24 h at 4 °C. After extensive rinsing with PBS, each sample was placed on a cover glass slide and air-dried at room temperature. The surface morphologies of fresh fat and AAM were observed using a scanning electron microscope (SEM; JSM-7401F; JEOL, Tokyo, Japan) after coating with platinum at an accelerating voltage of 15 kV. The AAM samples were fixed in 4% formalin, embedded in paraffin, sectioned into 4-mm slices, and affixed to glass slides. Hematoxylin and eosin (H&E) staining was performed to detect residual cells, nuclei, and adipocytes. Masson’s trichrome (MT) staining was used to detect collagen distribution.

#### In Vitro Cytotoxicity Tests

The in vitro cytotoxicity of AAM was evaluated according to the cell culture methods and exposure conditions described in the International Organization for Standardization 10993-5: Biological Evaluation of Medical Devices Test for Cytotoxicity: In Vitro Methods (ISO 10993-5:2009) [14].

##### Indirect Contact Test

The human keratinocyte line (HaCaT; Addexbio, San Diego, USA) underwent cultivation in Keratinocyte Growth Medium 2 (Promocell, Heidelberg, Germany), whereas the human foreskin fibroblast line (Hs27; ATCC® CRL-1634TM, Manassas, USA) was cultured in high-glucose Dulbecco’s modified Eagle’s medium (DMEM; Merck KGaA). Both culture media were supplemented with 10% inactivated fetal bovine serum (FBS; Invitrogen, Waltham, MA, USA) and 1% penicillin-streptomycin (Merck KGaA). These cultures were maintained at 37 °C within a 5% CO2 incubator. Subsequently, both cell types were passaged every 2–3 d with the introduction of fresh medium with the same composition. For the indirect contact method, the AAM extract was initially dissolved in a polar solvent and serially diluted 1:2 with fresh medium containing 10% FBS. The cells were then seeded into 96-well plates at a concentration of 1 × 104 cells per well and allowed to incubate for 24 h, maintaining a controlled environment at 37 °C and 5% CO2. Following the removal of the culture medium, the cells were incubated with 100 µL of the extract media for an additional 24 h. PBS-treated cells served as controls.

##### MTT Assay

Following thorough rinsing with PBS, each well received 20 µL of a 5 mg/mL solution of methyl thiazolyl tetrazolium (MTT; Roche, Switzerland), which was then allowed to incubate for a duration of 4 h at 37°C. Subsequently, a 200 µL volume of dimethyl sulfoxide (Merck KGaA) was introduced as the solubilization buffer for the extraction of formazan crystals. After 30 min of agitation on a plate shaker, the absorbance was measured at 570 nm using a microplate reader (VersamaxTM; Molecular Devices, San Jose, CA, USA). All experiments were conducted in triplicates. To evaluate cellular morphology, 1 × 105 cells per well were seeded into 12-well plates and cultured in the extract medium for 24 h. PBS-treated cells served as controls. Visual examination of cellular morphology was performed using an inverted compound light microscope (Leica, Wetzlar, Germany), and the corresponding cell images were documented.

##### Flow Cytometric Apoptosis Assay

Hs27 and HaCaT cells were seeded onto the sample substrates in 6-well plates. After incubation for 48 h, the test materials were carefully aspirated, and the cells were gently washed with PBS. The cells were detached using TrypLETM (Gibco), collected, and centrifuged at 1,300 rpm for 3 min. The resultant cell pellet was then resuspended in 500 µL of 1X Annexin V binding buffer. Subsequently, the cell suspensions were transferred into 5 mL round-bottom Falcon tubes, and 3 µL of FITC-annexin V, along with 5 µL of propidium iodide (PI), were introduced to facilitate staining. Each sample was divided into four distinct categories (unstained, FITC-annexin V-stained without PI, PI-stained without FITC-annexin V, and double-stained), thereby facilitating data normalization. The stained cells were incubated at room temperature for 30 min in the dark and subsequently subjected to analysis using the FACS Calibur system (BD, Franklin Lakes, USA). For each experimental run, data were acquired from 30,000 cells [15]. Cell culture media were used as controls. Following exposure to AAM for 48 h, apoptotic and necrotic cells were identified and quantified by flow cytometry.

### Acquisition of ASCs

For the primary culture of ASCs, inguinal adipose tissue was obtained from 7-week-old male C57BL/6J mice and digested with 0.2% collagenase type I (Worthington Biochemical Corp., Lakewood, USA) at 37°C for 1 h and centrifuged at 1,500 rpm for 3 min [16, 17]. The cell pellet was resuspended in DMEM/F-12, supplemented with GlutaMAX, 10% FBS, and 1% penicillin-streptomycin (Merck KGaA), and maintained at 37 °C in a 5% CO2 incubator. Cells were cultured to approximately 75% confluence, and passages 2–4 were used for all experiments.

### In Vivo AAM Transfer Analysis

#### In Vivo Volume Retention in a Mouse Model

A total of 24 eight-week-old male nude mice (Koatech Co., Seoul, Korea) were randomly divided into four different treatment groups (n=6 per group; Fig. S1): HA filler injection (HI), HA filler injection with 1×10^5^ ASCs (CHI), AAM transfer (AT), and AAM transfer with 1×10^5^ ASCs (CAT). In the CHI and CAT groups, ASCs were simultaneously grafted onto the scaffolds at the specified cell count. All groups were grafted with the same volume of 150 µL into the sub-panniculus muscle layer at the dorsal location through a 27G needle attached to a 0.5-mL syringe [18–20]. Under respiratory anesthesia, longitudinal measurement of the long axis of the grafted regions was conducted at weeks 4 and 8. Subsequently, all experimental animals were euthanized at week 8, and the grafted materials containing the regenerated tissue were meticulously retrieved. The collected specimens were weighed using an electronic scale.

All animal experiments were approved by the Institutional Animal Care and Use Committee (IACUC) of Seoul National University Hospital (IACUC No. 22-0167-S1A0(1)). All experiments were performed according to relevant guidelines and regulations, including ARRIVE. The animals were provided with food and water ad libitum.

#### Histological and IHC Analyses

At week 8 after grafting, the fixed specimens of the skin and subcutaneous tissue were analyzed by a pathological laboratory using H&E and MT stainings. An independent pathologist examined slides under a light microscope (Nikon ECLIPSE Ts2; Nikon, Tokyo, Japan). Images were captured using ImageJ software (National Institutes of Health, Bethesda, MD, USA). Subsequently, the mean number of adipocytes and percentage were calculated [21]. Using paraffin sections (4 µm) treated with deparaffinization and antigen retrieval, IHC staining was performed using an anti-CD31 antibody (1:100, PA5-16301, Invitrogen), anti-α smooth muscle actin (α-SMA; 1:100, ab5694, Abcam, Cambridge, UK) followed by incubation with horse-radish peroxidase-conjugated goat anti-mouse antibody (Dako, Glostrup, Denmark) and colorization with 3,3’-diaminobenzidine tetrahydrochloride (Dako). Six randomly selected discontinuous fields from each section were imaged using a light microscope (Eclipse 90i; NIKON Steel Co., Kawasaki, Japan). ImageJ was used to analyze the integrated optical density (IOD) of each stained sample. We selected 6 sections from each sample, specifically choosing sections that exhibited consistent staining patterns. The mean IOD values of the CD31 signals were calculated to assess vascularity. The mean IOD values of α-SMA signals were calculated to assess fibrosis [22, 23].

#### Gene Expression Analysis

The mRNA expression of different genes in the grafted materials and capsules was investigated at postoperative week 8. Total RNA was extracted using the RNeasy Lipid Tissue Mini Kit (Qiagen, Hilden, Germany). Reverse transcription was performed using TOPscript™ RT DryMIX (Enzynomics, Inc., Daejeon, Korea), and quantitative real-time polymerase chain reaction (PCR) was performed using the SYBR Green system (Enzynomics, Inc.). The housekeeping gene Gapdh was used as an internal reference. Interleukin 6 (Il6) is a pro-inflammatory cytokine that modulates the acute immune response to injury, whereas interleukin 10 (Il10) is an anti-inflammatory cytokine that plays a central role in limiting the host immune response to pathogens, thereby preventing host damage and maintaining normal tissue homeostasis [24, 25]. Transforming growth factor-β (TGF-β), a key mediator of fibrous capsular formation, recruits monocytes and induces fibroblast chemotaxis and differentiation into myofibroblasts [26]. The extent of revascularization was analyzed using vascular endothelial growth factor (Vegf) secretion, and peroxisome proliferator-activated receptor γ (Pparg) was used to analyze the degree of adipogenesis [19]. The primer sequences used in this study are listed in Table 1.

**Table 1:** Primer sequences for quantitative real-time polymerase chain reaction

Gene		Sequence
IL- 6	Forward	5′-ATAGTCCTTCCTACCCCAATTTCC-3′
	Reverse	5′-GATGAATTGGATGGTCTTGGTCC-3′
IL-10	Forward	5′- GACTTTAAGGGTTACCTGGGTTG-3′
	Reverse	5′- TCACATGCGCCTTGATGTCTG-3′
Tgf-β	Forward	5′- GGCCAGATCCTGTCCAAGC-3′
	Reverse	5′- GTGGGTTTCCACCATTAGCAC-3′
Vegf	Forward	5′-CATCTTCAAGCCGTCCTGTGT-3′
	Reverse	5′-CACTCCAGGGCTTCATCGTTA-3′
Pparg	Forward	5′- TGCACTGCCTATGAGCACTTCACA-3′
	Reverse	5′- AGGAATGCGAGTGGTCTTCCATCA-3′
Gapdh	Forward	5′-GCGACTTCAACAGCAACTCC-3′
	Reverse	5′-CCCTGTTGCTGTAGCCGTATT-3′

### Statistical Analysis

Statistical analyses were performed using IBM SPSS Statistics for Windows version 25 (IBM Corp., Armonk, NY, USA). The Mann–Whitney U or Kruskal–Wallis test was used to determine statistical significance between groups. Data are expressed as the mean ± standard deviation of the mean, and *p*-values <0.05 were considered statistically significant.

## Results

### Structure and Properties of AAM

**Fig. 2:**
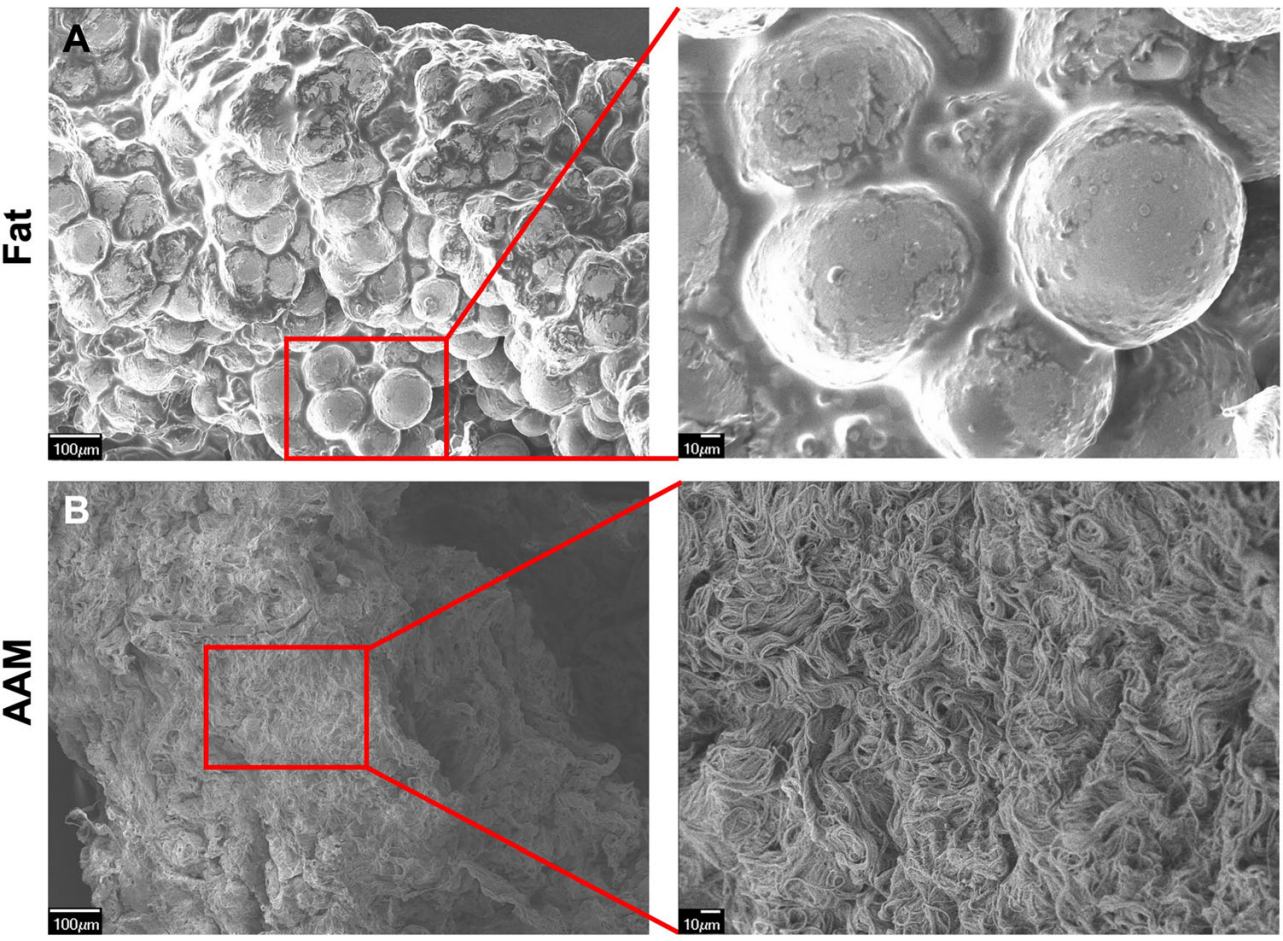
Structure of AAM and fat tissue. Scanning Electron Microscopy (SEM) images illustrating the structural differences between human adipose tissue and AAM. **A** SEM images of normal fat tissue. Fresh human adipose tissue exhibits multiple clusters comprising adipocytes, collagen fibers. **B** SEM images of AAM. manufactured without cellular components, displays well-distributed ultrastructures of network-type collagen fibers. The structure of the collagen fiber network is evenly distributed in the AAM. Scale bars, 100×: 100 µm, 400×: 10 µm.

**Fig. 3:**
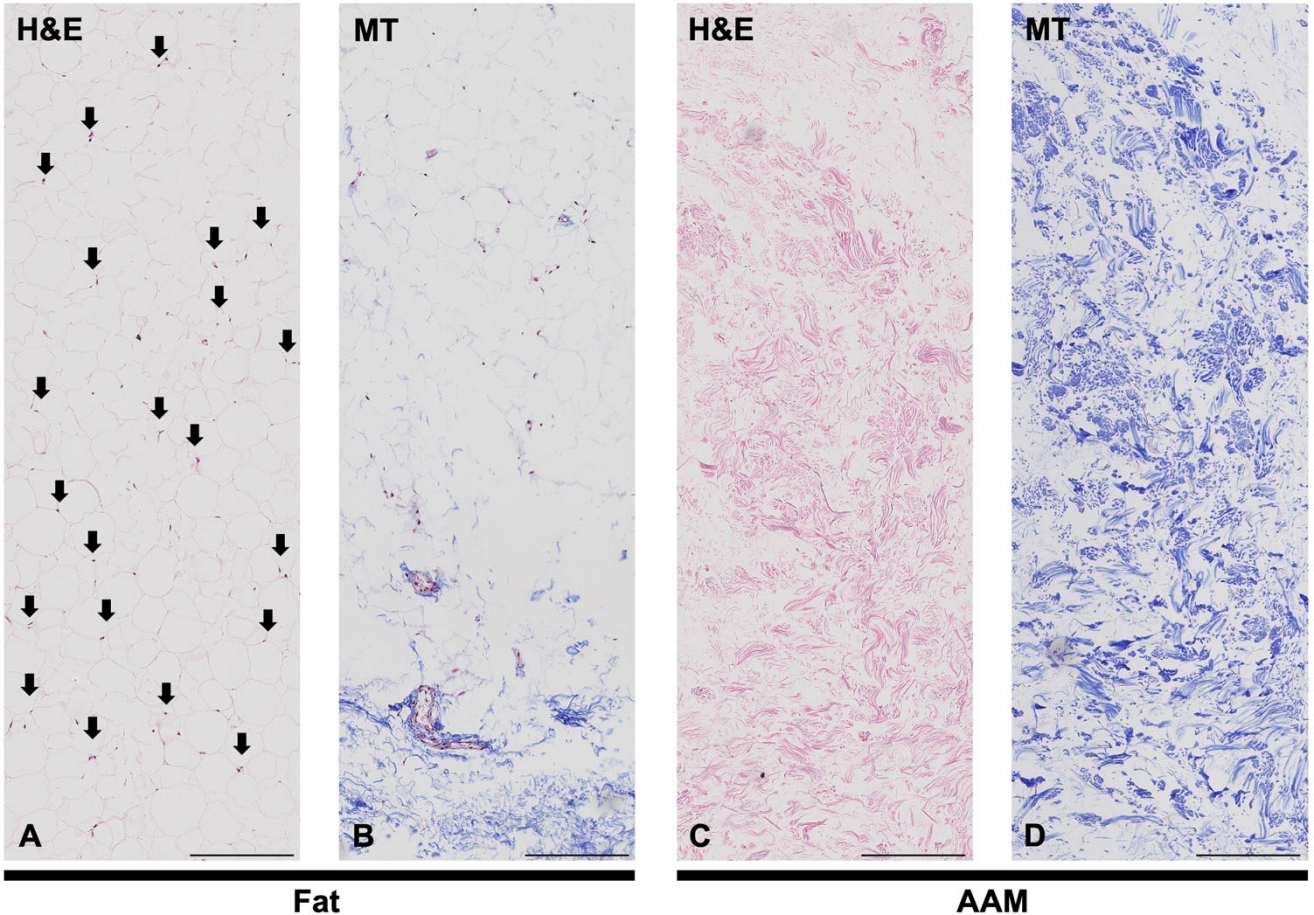
Components of AAM and fat tissue. H&E staining confirmed the efficacy of our decellularization protocol, with no residual cells or debris present in the AAM (Fig. 3A, 3C). MT staining also showed uniformly distributed bundles of acellular collagen fibers of varying thickness in the AAM (Fig. 3B, 3D). **A** H&E-stained normal human fat tissue. Black arrows indicate cell nuclei present in the adipose tissue. **B** MT-stained normal human fat tissue. **C** H&E-stained AAM. Only collagen fiber bundles with no residual cells are visible, confirming the effectiveness of the decellularization process. **D** MT-stained AAM. MT staining shows an evenly distributed collagen organization in the AAM sample. Scale bars, 200 µm. *AAM* acellular adipose matrix, *H&E* hematoxylin and eosin, *MT* Masson’s trichrome, *SEM* scanning electron microscope. Black arrows adipocyte cell nucleus

The gross appearance of human adipose tissue had a mixed yellow and red color, whereas AAM contained only whitish fibrotic tissues (Fig. S2). In SEM images, multiple clusters containing adipocytes, collagen fibers, endothelial cells, and pericytes were observed in the fresh human adipose tissue (Fig. 2A). Only well-distributed ultrastructures of network-type collagen fibers were observed in the AAM manufactured without cellular components (Fig. 2B). H&E staining confirmed the efficacy of our decellularization protocol, with no residual cells or debris present in the AAM (Fig. 3A, 3C). MT staining also showed uniformly distributed bundles of acellular collagen fibers of varying thickness in the AAM (Fig. 3B, 3D).

### In Vitro Cytotoxicity of AAM

**Fig. 4:**
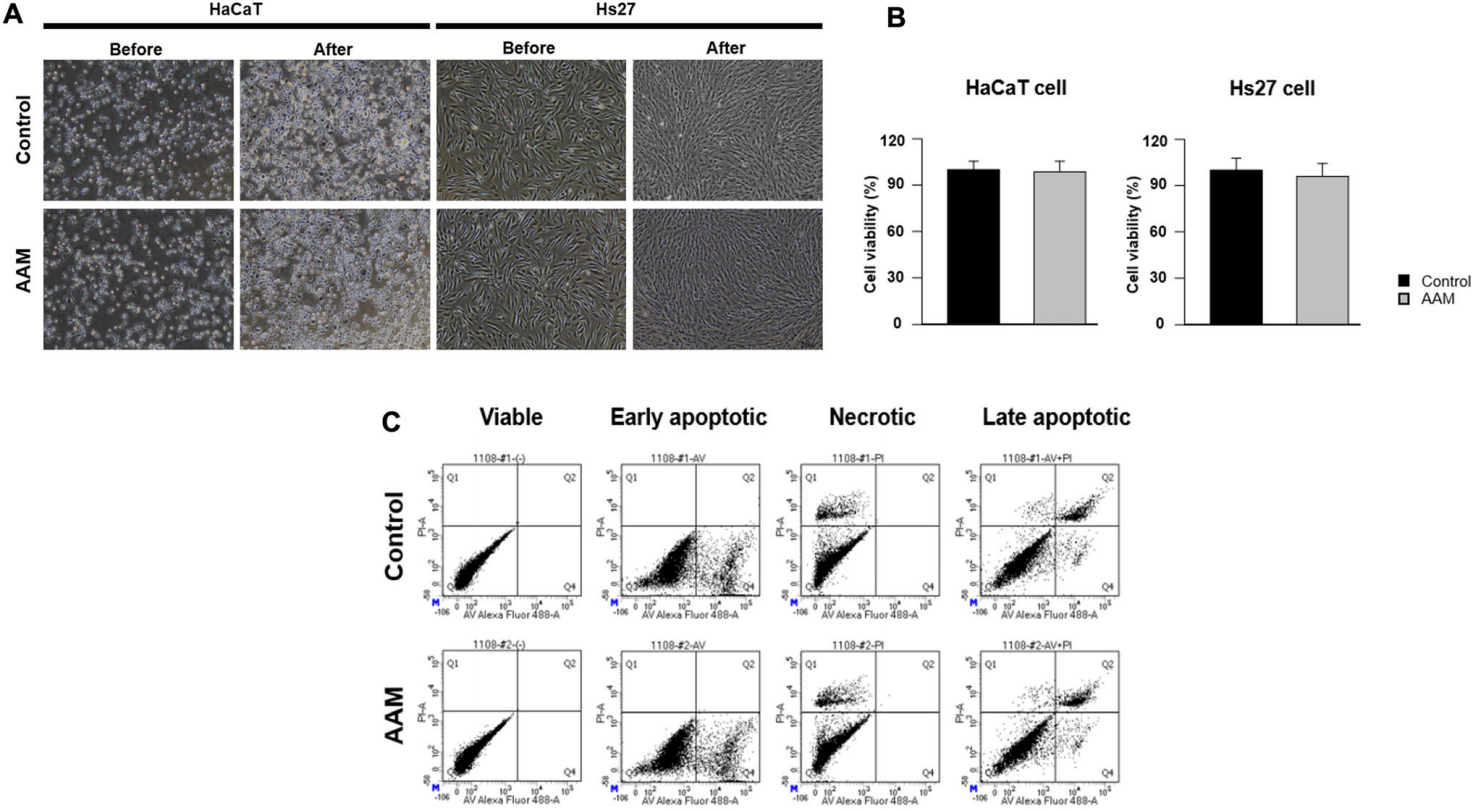
In vitro cytotoxicity of AAM. Morphological and viability assessments of Hs27 and HaCaT cell lines in response to AAM exposure. **A** Light microscopy images reveal that Hs27 cells exhibit a polygonal spindle shape, characteristic of fibroblast morphology, while HaCaT cells display a typical epidermal cell shape resembling cobblestones. No morphological differences were observed between AAM-conditioned media and control media for both cell lines, indicating that AAM did not alter human skin cell morphology. Scale bars, 100 µm. **B** MTT assay results show no difference in cell viability of Hs27 and HaCaT cells between AAM and control conditions (HaCaT Control: median 108.0%; HaCaT AAM: 106.0%; Hs27 Control: 103.0%; Hs27 AAM: 98.0%; n=18), indicating that AAM did not induce cytotoxicity in either cell line. **C** Flow cytometry analysis shows no difference in the ratio of apoptotic and necrotic cells between the AAM and control conditions. The live cell populations are shown in the lower left quadrants, and the cells in the early stages of apoptosis are in the lower right quadrants. The upper left quadrants show dead cells, and the upper right quadrants indicate apoptotic and necrotic cells. *FL3-H* propidium iodide, *FL1-H* FITC-annexin V, *AAM* acellular adipose matrix, *MTT* methyl thiazolyl tetrazolium

In the indirect contact method, Hs27 cells showed a polygonal spindle shape, a typical fibroblast morphology, whereas HaCaT cells showed the standard epidermal cell shape resembling cobblestones (Fig. 4A). No morphological differences in light microscopy images were found between AAM-conditioned and control media in both HaCaT and Hs27 cell lines, indicating that AAM did not affect human skin cells. In the MTT assay, no significant differences in cell viability were observed between the control and AAM groups in either cell line (HaCaT Control: median 108.0%; HaCaT AAM: 106.0%; Hs27 Control: 103.0%; Hs27 AAM: 98.0%; n=18), indicating that AAM did not induce cytotoxicity in either cell line (Fig. 4B). In the flow cytometry analysis, the ratio of apoptotic to necrotic cells did not significantly differ between the control and AAM groups (annexin V Control: 20.1%, annexin V AAM: 21.4%, n=6; PI Control: 12.3%, PI AAM: 12.8%, n=6; Fig. 4C). These results showed that AAM had no cytotoxic effects in vitro.

### In Vivo Volume Retention in a Mouse Model

**Fig. 5:**
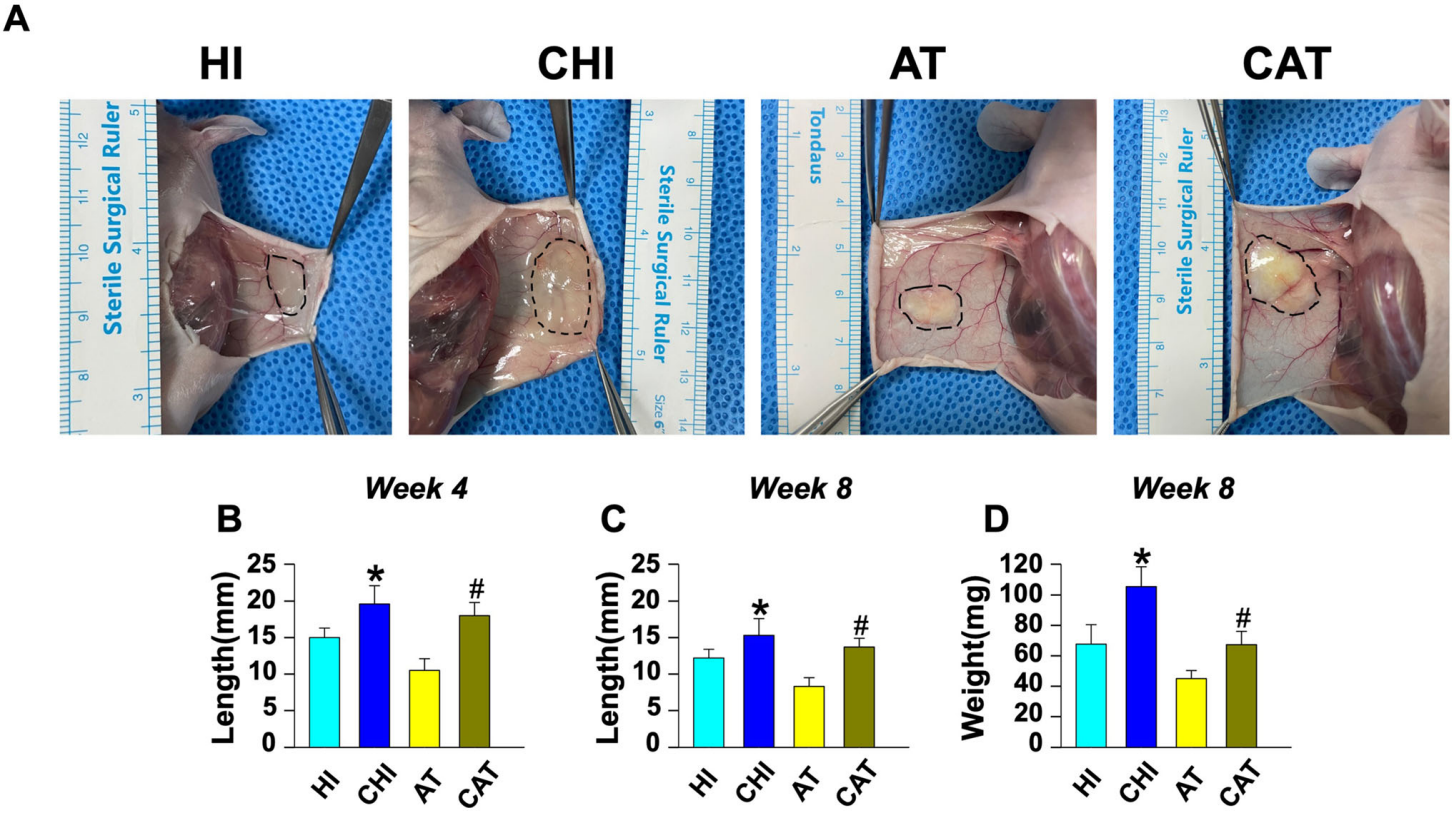
In vivo volume retention in a nude mouse graft model. Nude mice were grafted with HA filler injection (HI), HA filler injection with ASCs (CHI), AAM transfer (AT), or AAM transfer with ASCs (CAT). At postoperative week 8, in vivo graft materials were found with relatively well-circumscribed boundaries in all groups. Neovascularization was observed particularly in CAT. **A** Gross appearance of the grafted material at the dorsum of nude mice photographed at week 8. The black dotted lines indicate the well-circumscribed borders of grafted material (HA filler (HI), HA filler with ASCs (CHI), AAM (AT), and AAM with ASCs (CAT)). **B** The length of the longest axis of grafted material was significantly longer in CHI compared to HI group (*p<0.05), and in CAT compared to AT (#*p*<0.05) at week 4. **C** At week 8, the length of the longest axis of grafted material was also significantly longer in CHI compared to HI (*p<0.05), and in CAT compared to AT (#*p*<0.05). **D** The weight of the grafted material measured at week 8 was significantly greater in CHI compared to HI (*p<0.05) and in CAT compared to AT (#*p*<0.05). *AAM* acellular adipose matrix, *ASC* adipose-derived stem cell, *HA* hyaluronic acid

Nude mice were grafted with HA filler injection (HI), HA filler injection with ASCs (CHI), AAM transfer (AT), or AAM transfer with ASCs (CAT). At postoperative week 8, in vivo graft materials were found with relatively well-circumscribed boundaries in all groups. Neovascularization was observed particularly in CAT (Fig. 5A). The longest axis of the graft material measured at week 4 was significantly longer in CHI than in HI, and in CAT than in AT (HI: median [interquartile range] 15.5mm [14–16], CHI: 20.0mm [18–21], *p*<0.05; AT: 10.5mm [10, 11], CAT: 17.5mm [17–19], CHI vs. HI, *p*<0.05; CAT vs. AT, *p*<0.05; Fig. 5B). Likewise, the graft material was also at week 8 significantly longer in CHI than in HI, and in CAT than in AT (HI: 12.0mm [11–13], CHI: 15.0mm [13–17], *p*<0.05; AT: 8.5mm [7–9], CAT: 13.5mm [13–15], CHI vs. HI, *p*<0.05; CAT vs. AT, *p*<0.05; Fig. 5C). The weight of the graft material measured at week 8 was highest in CHI, followed by HI, CAT, and AT (HI: 70.0mg [54–79], CHI: 103.0mg [98–112], *p*<0.05; AT: 44.0mg [2, 42–48], CAT: 65.0mg [61–73]; Fig. 5D). The weight was significantly higher in CHI than in HI (*p*<0.05), and in CAT than in AT (*p*<0.05), whereas CAT maintained weight comparable to HI.

### Histological and IHC Analyses

**Fig. 6:**
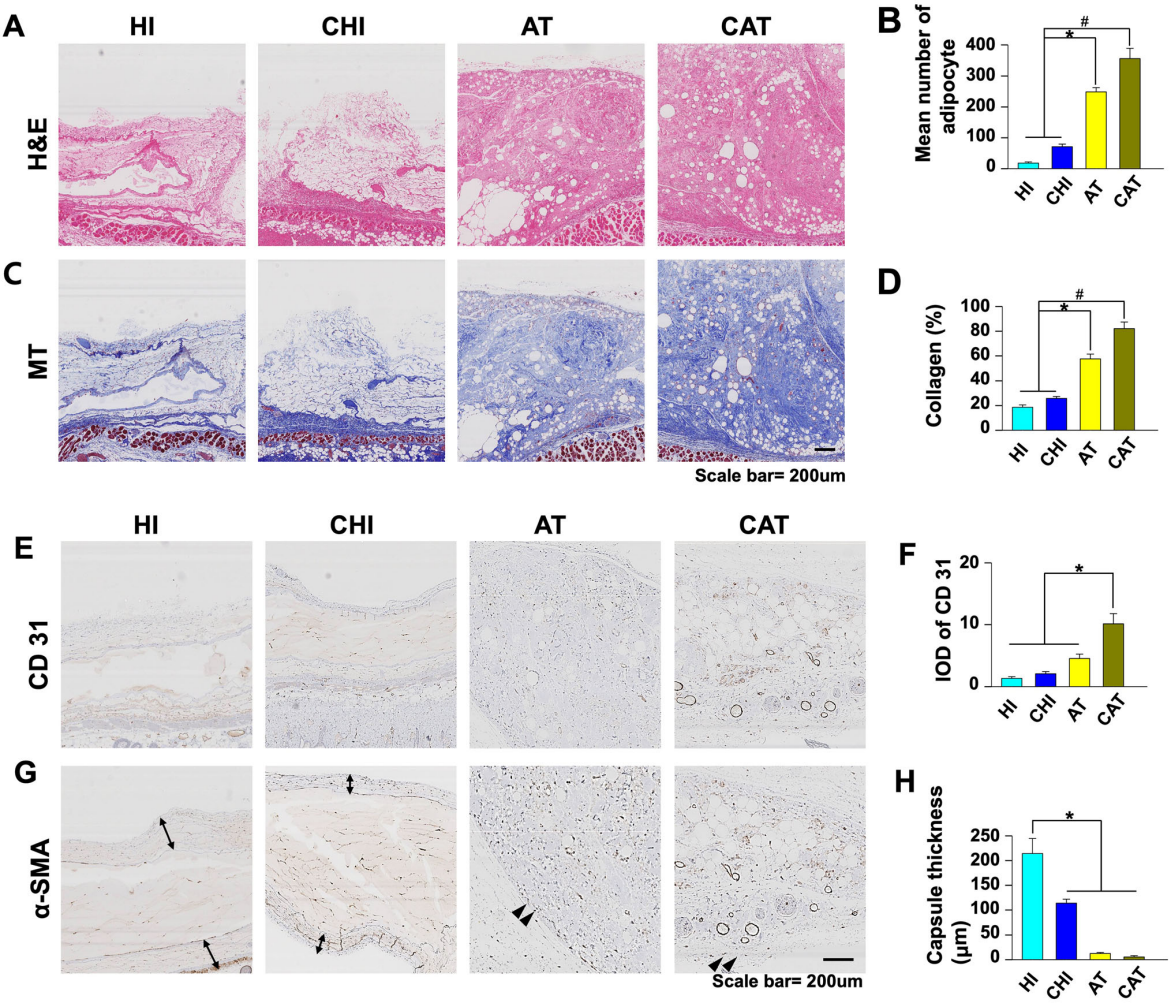
Histological and immunohistochemical analyses. **A**, **B** The mean adipocyte count in H&E stainings at week 8 was significantly higher in AT (*p<0.05, vs. HI, CHI) and CAT (#*p*<0.05, vs. HI, CHI) than in HI and CHI. **C**, **D** The mean area percentage of collagen in MT-stained slices at week 8 showed significantly higher ratios in AT (*p<0.05, vs. HI, CHI) and CAT (#*p*<0.05, vs. HI, CHI) groups than in HI and CHI. **E**, **F** At week 8, the IOD of the vascularity marker CD31 was significantly higher in CAT than in the other three groups (*p<0.05, vs. HI, CHI, AT). **G**, **H** Capsule thickness, semiquantitatively evaluated by α-SMA staining, was at week 8 significantly greater in HI than in the other three groups (*p<0.05, vs. CHI, AT, CAT) and almost absent in AT and CAT. *H&E* hematoxylin and eosin, *MT* Masson’s trichrome, *IOD* integrated optical intensity, *α-SMA* α smooth muscle actin, *AT* acellular adipose matrix transfer, *CAT* acellular adipose matrix transfer with ASCs, *HI* hyaluronic acid filler injection, *CHI* hyaluronic acid filler injection with ASCs, Black arrows capsule, Double black arrow: capsule (AAM-dermis junction), Scale bar= 200um

The median number of adipocytes in H&E staining at week 8 was significantly higher in AT and CAT than in HI and CHI (HI: 17.5 [17, 18], CHI: 72 [68–76], AT: 252 [247–257], CAT: 360.5 [358–363], AT vs. HI, CHI, *p*<0.05; CAT vs. HI, CHI, *p*<0.05; Fig. 6A, 6B). The mean percentage of collagen in MT-stained sections from week 8 was also significantly higher in AT and CAT than in HI and CHI (HI: 18.4% [18.2–18.5], CHI: 25.8% [25.3–26.3], AT: 57.5% [56.6–58.4], CAT: 84.4% [83.5–85.2], AT vs. HI, CHI, *p*<0.05; CAT vs. HI, CHI, *p*<0.05; Fig. 6C, 6D). These results show a higher deposition of adipocytes and collagen tissue in the AAM groups (AT and CAT) than in HA groups (HI and CHI).

The CD31 IODs at week 8 were significantly higher in CAT compared to the other three groups, indicating that cell-assisted AAM promoted the greatest tissue neovascularization (HI: 4.3 [4.2–4.3], CHI: 6.7 [5.7–6.7], AT: 11.2 [9.6–11.2], CAT: 14.8 [14.5–14.8], *p*<0.05; Fig. 6E, 6F). In semiquantitative analyses, the α-SMA-stained area at week 8, indicating the amount of capsule formation, was significantly greater in HI compared to the other three groups and almost absent in AT and CAT (HI: 201.4µm [192.2–210.6], CHI: 111.1µm [106.7–107.3], AT: 12.15 µm [11.2–13.1], CAT: 4.7 µm [4.5–4.9], *p*<0.05; Fig. 6G, 6H).

### Gene Expression Analysis

**Fig. 7:**
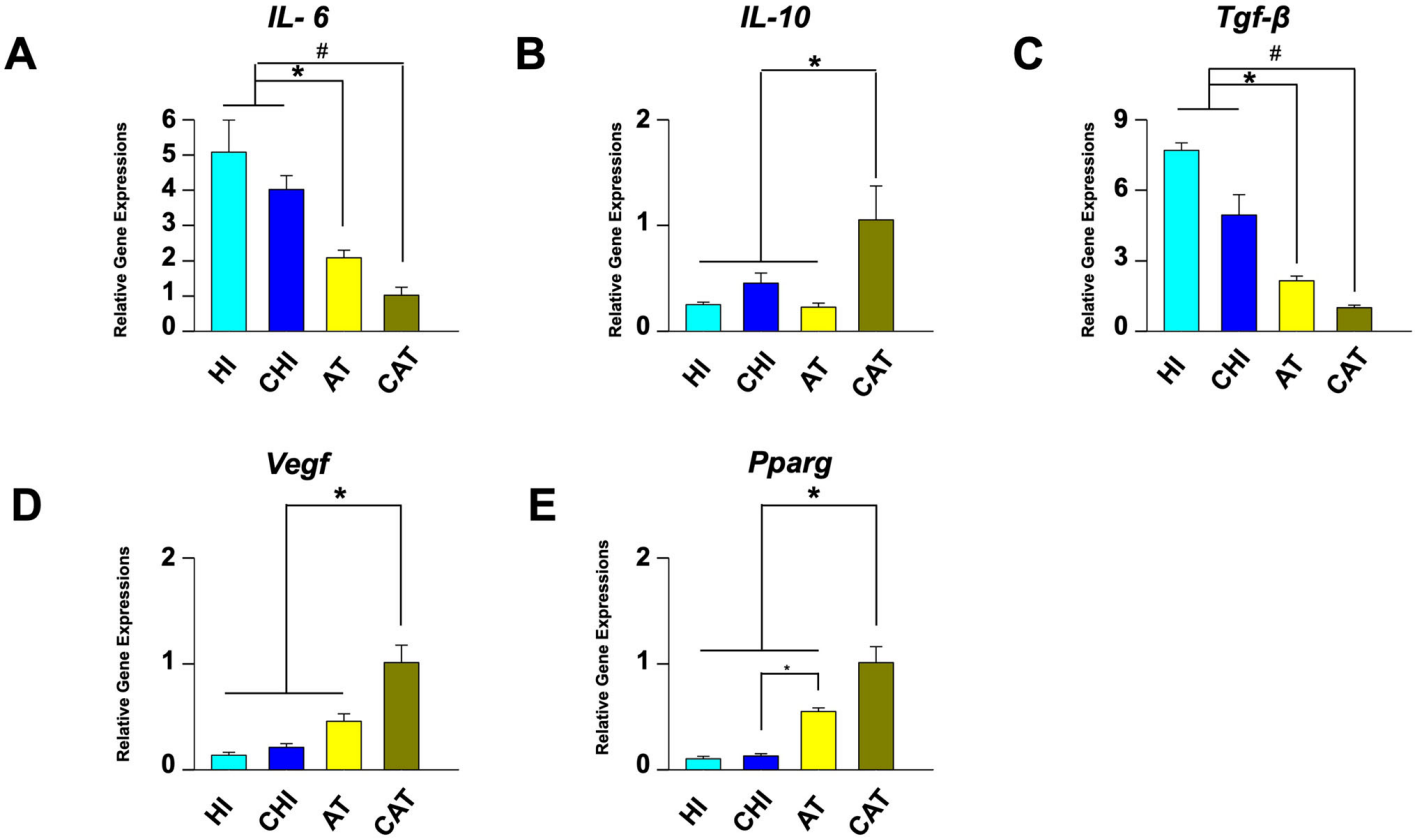
Analysis of mRNA expression level by RT-PCR. The enhanced angiogenic and adipogenic potential of cell-assisted AAM was demonstrated by elevated expression levels of Pparg and Vegf, particularly noted in the CAT group. This suggests that the combination of AAM and ASCs may be more effective for soft tissue regeneration than AAM alone. Specifically, the pro-inflammatory cytokine IL-6 levels were significantly lower in both AT and CAT compared to HI and CHI, while the anti-inflammatory cytokine IL-10 was significantly higher in CAT. Additionally, TGF-β levels, a key mediator of fibrous capsule formation, were significantly reduced in AT and CAT relative to HI and CHI. Vegf expression was notably higher in CAT compared to the other groups, and Pparg levels, crucial for adipogenesis, were significantly elevated in CAT, with AT also showing higher levels than CHI. These findings collectively underscore the potential of AAM combined with ASCs in promoting tissue regeneration. **A** Il-6 expression was significantly lower in AT (*p<0.05, vs. HI, CHI) and CAT (#*p*<0.05, vs. HI, CHI) groups than in HI and CHI. **B** Il-10 levels were significantly higher in CAT (*p<0.05, vs. HI, CHI, AT) than in HI, CHI, and AT. **C** The TGF-β levels were significantly lower in AT (*p<0.05, vs. HI, CHI) and CAT (#*p*<0.05, vs. HI, CHI) than in HI and CHI. **D** Vegf was significantly higher in CAT than in the other three groups (*p<0.05, vs. HI, CHI, AT). **E** Pparg expression was significantly higher in CAT compared to the other three groups. (*p<0.05, vs. HI, CHI, AT). *AT* acellular adipose matrix transfer, *CAT* acellular adipose matrix transfer with ASCs, *HI* hyaluronic acid filler injection, *CHI* hyaluronic acid filler injection with ASCs, *Il-6* interleukin 6, *Il-10* interleukin 10, *Pparg* peroxisome proliferator-activated receptor γ, *TGF-β* transforming growth factor, *Vegf* vascular endothelial growth factor

The expression levels of the pro-inflammatory cytokine Il-6 were significantly lower in AT and CAT than in HI and CHI (HI: median 4.6, CHI: 3.79, AT: 2.12, CAT: 0.92; HI, CHI vs. AT, *p*<0.05; HI, CHI vs. CAT, *p*<0.05; Fig. 7A). The expression of Il-10, an anti-inflammatory cytokine, was significantly higher in CAT than in the other three experimental groups (HI: 0.27, CHI: 0.51, AT: 0.20, CAT: 1.06; *p*<0.05; Fig. 7B). The levels of TGF-β, a key mediator of fibrous capsule formation, were significantly lower in AT and CAT compared to HI and CHI (HI: 7.26, CHI: 5.41, AT: 2.19, CAT: 0.99; HI, CHI vs. AT *p*<0.05; HI, CHI vs. CAT *p*<0.05; Fig. 7C). The expression of Vegf, a vascular marker, was significantly higher in CAT than in the other three groups (HI: 0.13, CHI: 0.18, AT: 0.49, CAT: 0.91; CAT vs. HI, CHI, and AT, *p*<0.05; Fig. 7D). The expression of Pparg, which plays a key role in adipogenesis, was significantly higher in CAT than in the other three groups (Fig. 7E). In addition, the Pparg level in AT was significantly higher than that in CHI (HI: 0.11, CHI: 0.12, AT: 0.53, CAT: 11.06; CAT vs. HI, CHI, AT, *p*<0.05; AT vs. CHI, *p*<0.05).

## Discussion

In the need for a biomaterial solution that can provide the biological benefits of autologous adipose tissue and the ease of use of HA fillers, AAM has attracted much attention because of its wide range of sources and good volume repair capacity [8]. A previous proteomic analysis revealed that AAM is composed of collagens (I, III, IV, VI, and VII), glycosaminoglycans, laminin, elastin, fibronectin, and growth factors such as VEGF and fibroblast growth factor 2 [4]. It is crucial to decide how to manufacture AAM because the decellularization process greatly affects its components, structural and functional characteristics, thus determining the efficacy in regenerative tissue engineering [27, 28]. Currently, a universally accepted standardized protocol for adipose tissue decellularization does not exist. The optimal method involves processing discarded fat through a series of chemical, mechanical, and enzymatic treatments to remove cells, cellular components, and lipids and yield the final product of a 3D bioscaffold that mimic the biochemical properties of the native extracellular matrix (ECM) [29].

In the comprehensive review by Sano et al., the protocol described by Flynn was the most efficient method for both decellularization and maintaining the native ECM constituents [30]. While other methods employ detergents such as SDS or Triton X-100, which may interact with ECM components and become cytotoxic, the protocol by Flynn avoids the use of detergents and preserves the AAM key structures [12, 31]. Thus, our study used a modified version of Flynn’s protocol to preserve the unique features of adipose tissues, including their proteomic composition and rheological properties, and to remove cellular components that can induce immune reactions. It was modified by maximizing the number of freeze-thaw cycles to minimize the following chemical processes: the DNase and RNase doses, polar solvent extraction time, and incubation time of each step. Minimizing the dose of chemical ingredients can improve biocompatibility, and reducing the total processing time can be beneficial regarding the cost and scalability of the approach.

The efficacy of our decellularization protocol was confirmed by observing SEM, H&E-stained, and MT-stained images, in which the 3D ultrastructures of collagen fibers were well distributed without any residual cells while resembling the native ECM environment. The in vitro cytotoxicity of the manufactured AAM was evaluated using an indirect contact test, MTT assay, and flow cytometric analysis and proved its safety.

Numerous studies have examined AAM as a scaffold or an inductive template for reconstructive tissue remodeling [32–37]. However, few studies have focused on its potential as a stand-alone volume substitute for commercially available HA fillers. Young et al. reviewed and compared the use of commercially available soft tissue fillers and ECM-based biomaterials for adipose tissue engineering and found that ECM-based products have better potential to promote de novo adipogenesis and hence can promote long-term volume retention [38]. However, this was a review article, and no preclinical or clinical experiments directly comparing the volume retention of AAM and HA have been reported.

In the current study, the in vivo volume retention of AAM and HA was directly compared. Although the AAM groups (AT and CAT) exhibited lower volume retention than the HA groups (HI and CHI) at week 8, CAT showed a similar retention as HI, indicating that the volumizing effect of cell-assisted AAM is comparable to that of HA without cellular assistance. Moreover, histological analysis revealed a significantly higher adipocyte count and collagen formation in the AAM groups than in the HA groups. These phenomena can be attributed to the better mechanical support of the remaining HA filler material and its biochemical properties to retain substantial water from the surrounding tissues, resulting in greater volume preservation compared to the AAM groups at week 8. However, this volume would eventually undergo degradation in the long term [39]. In contrast, the AAM groups exhibited conspicuous augmentation of adipogenic regeneration and maintenance of injected collagen, as well as the genesis of novel collagen tissues. Fundamentally, although the AAM groups may trail behind the HA groups in volume preservation at week 8, they concurrently manifest the capability to regenerate adipose/collagen tissue and maintain volume in the long term.

In general, two theories describe the physiology of fat graft outcomes: the partial survival of grafted adipocytes and the potential ability of the remaining stromal cells to recruit host tissues to fill the void through neovascularization, adipogenesis, and fibrosis [1, 3]. Similarly, in AAM, biological cues from the ECM, including growth factors and proteins, are known to promote host cell migration, vascularization, and tissue development within the implanted acellular scaffolds, leading to the formation of new viable tissue with potential permanence [8]. These features of neovascularization and adipogenesis are not typically associated with synthetic scaffolds, which makes AAM a biomaterial superior to HA regarding tissue regeneration [2, 40–42]. Consequently, it is evident that more long-term observational studies focusing on volume retention and tissue regeneration of AAM are imperative for future research.

Regarding immune reaction and biocompatibility, the Il-6 level was significantly higher in the HA groups than in the AAM groups, whereas the Il-10 level was significantly lower. These findings indicate that the level of inflammation was decreased in the AAM groups through the downregulation of Il-6, a gene that usually leads to fibrosis and scarring through M1 macrophage activation and upregulated expression of MHC class II antigens. The simultaneous upregulation of M2 phase-related Il-10 expression in the AAM groups suppressed inflammation and promoted matrix remodeling and repair [43, 44]. Consequently, the level of TGF-β expression and the thickness of the fibrous capsule evaluated by α-SMA staining were both much greater in the HA groups than in the AAM groups. In particular, the AAM groups exhibited almost no capsule formation. This demonstrates the better biocompatibility of the AAM-based grafts compared to that of the HA fillers.

AAM showed better performance in terms of volume retention and tissue regeneration when supplemented with ASCs. The volume retention at week 8 was significantly greater in CAT than in AT. IHC staining showed the highest vascularity (CD31+) in CAT, followed by AT and HA-based groups, and mRNA gene expression analysis showed the highest level of vascularization-related (Vegf) and adipogenesis-related (Pparg) genes in CAT. Although the definite superiority of cell-assisted AAM transfer over AAM implantation alone remains unclear, numerous studies have examined AAM as a scaffold for ASC supplementation to enhance long-term angiogenesis and adipogenesis in vivo [27, 28, 34, 37, 38, 45, 46]. Poon et al. reported that AAM alone stimulated adipogenesis equivalent to 30% of the original volume grafted after 8 weeks in rats, compared to 48% when grafted with supplemented ADSCs [47]. Zhang et al. studied the potential of AAM with ASCs in mice and reported significant adipogenesis and neovascularization within 6–12 weeks, whereas AAM alone induced adipogenesis to a lesser extent and was almost completely absorbed after 12 weeks [48].

The ASCs supplemented in the AAM scaffolds are known to provide an inductive microenvironment for adipogenesis, expressing high levels of chemotactic, proangiogenic, and immunomodulatory factors, including PPARγ and C/EBPα, the major regulators of adipogenesis [30, 46, 47, 49]. This indicates that AAM plays an important role in providing a cell-supportive delivery platform for ASCs and mediating adipogenic differentiation [50, 51]. In addition, ASCs contribute to fat tissue regeneration by triggering the migration of host stem cells to the site of implantation and promoting vascularization, which is essential for long-term fat stability [52–54]. In our study, the improved angiogenic and adipogenic potential of cell-assisted AAM was proven by the high expression levels of Pparg and Vegf, as well as the high vascularity, adipocyte count, and collagen deposition, in the CAT group. These results suggest that the combination of AAM and ASCs may be more effective for soft tissue regeneration than AAM alone [46, 47, 49]

Overall, the results of this study suggest that cell-assisted AAM is capable of retaining a volume comparable to that of HA filler and has a much greater potential for tissue regeneration than HA filler or AAM alone. Therefore, it is considered an ideal biomaterial. However, as a limitation of this approach, cell-assisted AAM has the fundamental disadvantage of requiring invasive liposuction to obtain ASCs. Although AAM implantation alone showed a lower volume retention rate than HA filler in relatively short-term follow-up (8 weeks), it showed greater potential for tissue regeneration and better biocompatibility than HA filler. Because AAM has definite potential in angiogenesis, adipogenesis, and collagen deposition owing to the inductive properties of the ECM to recruit host cells and facilitate de novo adipogenesis, positive results in volume restoration are expected in more long-term follow-ups. With further related studies, the use of AAM transfer could provide a major advantage to current fat grafting, as it obviates the need for autologous fat harvesting while using natural human adipose-derived scaffolds as volume substitutes. Further studies on the use of AAM supplemented with platelet-rich plasma, which does not require liposuction, also could be considered. Moreover, the anticipated long-term advantages of AAM over HA fillers warrant a multifaceted approach to future research. By pursuing comparative clinical trials, large-volume injection studies, longitudinal follow-up, mechanistic studies, and incorporating patient-reported outcomes, the clinical community can gain deeper insights into AAM’s benefits and limitations. This comprehensive understanding will ultimately pave the way for enhanced patient care and satisfaction in aesthetic and reconstructive medicine. Finally, as this study compared volume retention and tissue restoration between AAM and HA fillers, a comparison between AAM and conventional fat grafting could be performed.

In conclusion, AAM transfer supplemented with ASCs resulted in volume augmentation comparable to that of the HA filler. With its angiogenic and adipogenic abilities and good biocompatibility, AAM has the potential to be an off-the-shelf filler substitute without the need for invasive liposuction, efficiently utilizing the vast amount of discarded fat tissues. If officially approved in the future, AAMs could be applied in various forms and become a more physiological option for soft tissue reconstruction than currently used acellular dermal matrices. Cell-assisted AAM transfer is a promising technique for volume retention and tissue regeneration, offering a safe and effective alternative to HA filler injection.

## Supplementary Information

Below is the link to the electronic supplementary material.

**Supplementary Fig. S1.** Study design for in vivo volume retention in a nude mouse model. *HI* hyaluronic acid filler injection, *CHI* hyaluronic acid filler injection with ASCs, *AT* acellular adipose matrix transfer, *CAT* acellular adipose matrix transfer with ASCs, *HA* hyaluronic acid, *ASC* adipose-derived stem cell, *AAM* acellular adipose matrix (JPEG 358 KB)

**Supplementary Fig. S2.** Gross appearance of human adipose tissue (**A**) and the final acellular adipose matrix (**B**) (JPEG 724 KB)

## Data Availability

The data used during the current study are available from the corresponding author on a reasonable request.
