# Supplementary figures and images for: Efficacy and Safety of Cell-Assisted Acellular Adipose Matrix Transfer for Volume Retention and Regeneration Compared to Hyaluronic Acid Filler Injection
Source: Aesthetic Plast Surg. 2024 Oct 1;49(5):1276–89. doi: 10.1007/s00266-024-04408-0 (PMC11965223; doi:10.1007/s00266-024-04408-0)

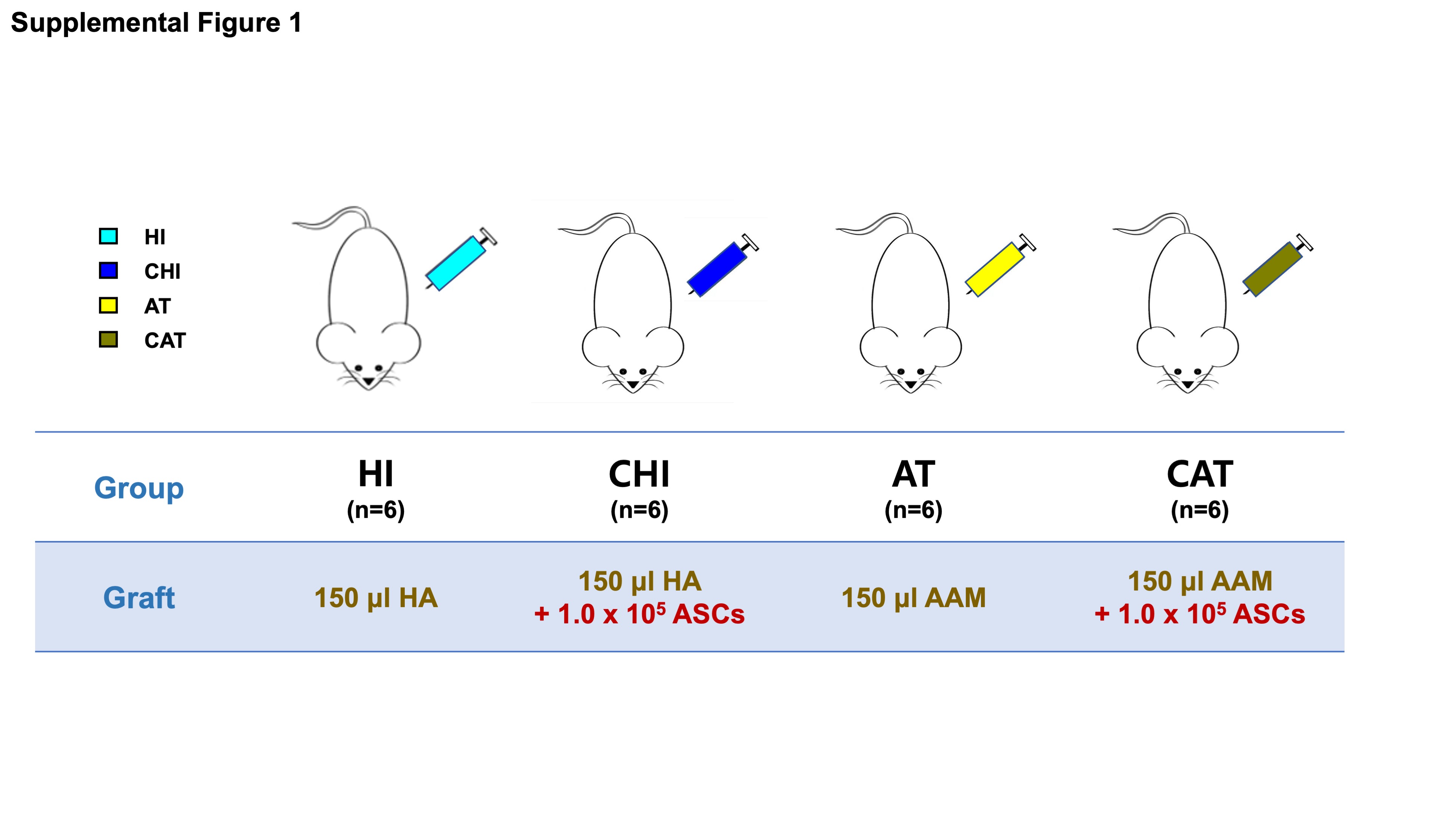

Supplement: Supplementary file 1 — Supplementary Fig. S1. Study design for in vivo volume retention in a nude mouse model. HI hyaluronic acid filler injection, CHI hyaluronic acid filler injection with ASCs, AT acellular adipose matrix transfer, CAT acellular adipose matrix transfer with ASCs, HA hyaluronic acid, ASC adipose-derived stem cell, AAM acellular adipose matrix (JPEG 358 KB) [file 266_2024_4408_MOESM1_ESM.jpeg]

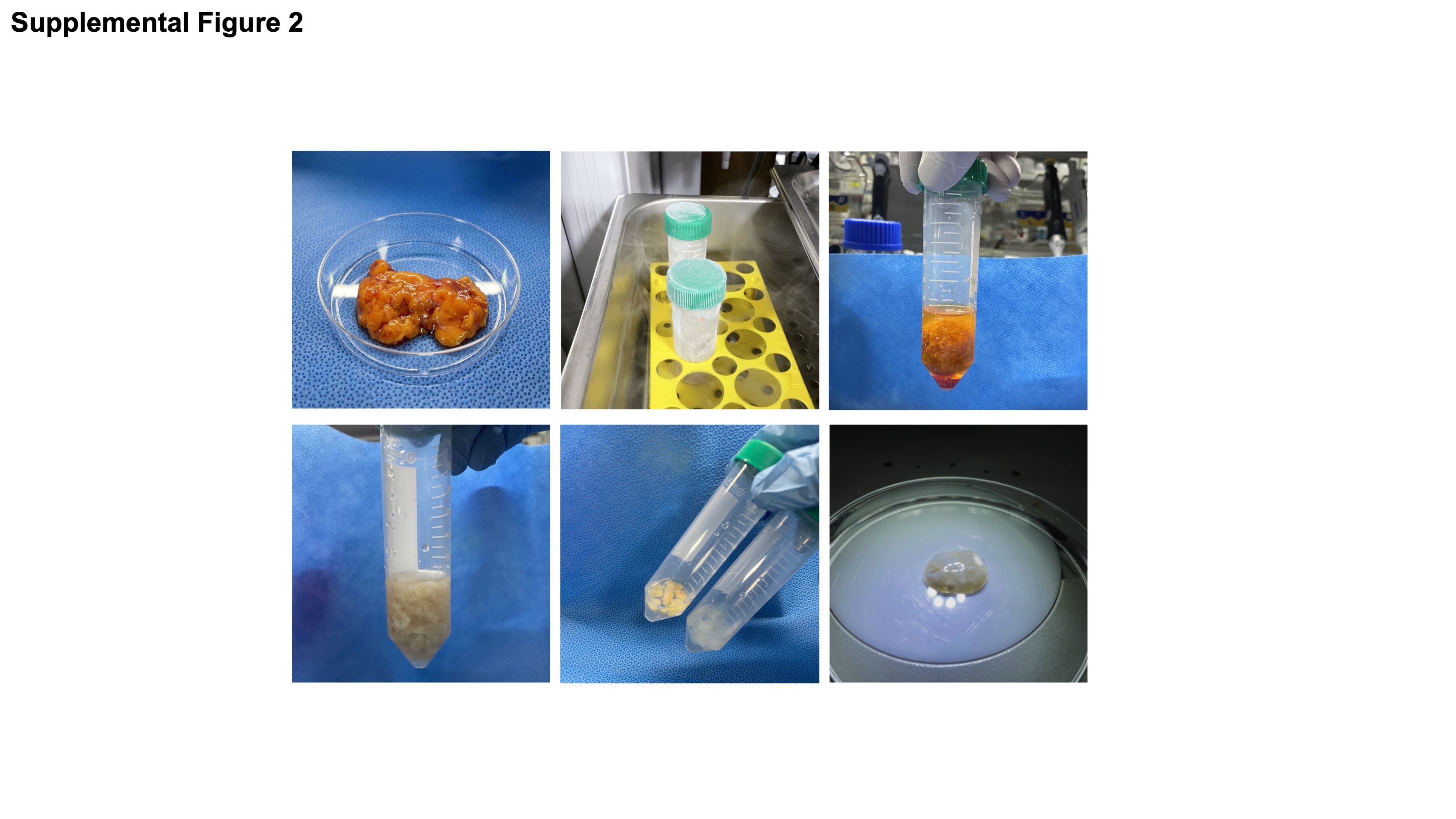

Supplement: Supplementary file 2 — Supplementary Fig. S2. Gross appearance of human adipose tissue (A) and the final acellular adipose matrix (B) (JPEG 724 KB) [file 266_2024_4408_MOESM2_ESM.jpeg]
